# An Association between Air Pollution and Daily Outpatient Visits for Respiratory Disease in a Heavy Industry Area

**DOI:** 10.1371/journal.pone.0075220

**Published:** 2013-10-25

**Authors:** Kuo-Ying Wang, Tang-Tat Chau

**Affiliations:** 1 Department of Atmospheric Sciences, National Central University, Chung-Li, Taiwan; 2 Department of Community Medicine, Taiwan Landseed Hospital, Ping-Jen, Taiwan; University of Liverpool, United Kingdom

## Abstract

In this work we used daily outpatient data from the Landseed Hospital in a heavily industrial area in northern Taiwan to study the associations between daily outpatient visits and air pollution in the context of a heavily polluted atmospheric environment in Chung-Li area during the period 2007–2011. We test the normality of each data set, control for the confounding factors, and calculate correlation coefficient between the outpatient visits and air pollution and meteorology, and use multiple linear regression analysis to seek significance of these associations. Our results show that temperature and relative humidity tend to be negatively associated with respiratory diseases. NO and 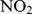 are two main air pollutants that are positively associated with respiratory diseases, followed by 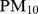, 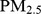, 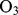, CO, and 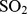. Young outpatients (age 0–15 years) are most sensitive to changing air pollution and meteorology factors, followed by the eldest (age 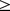
66 years) and age 16–65 years of outpatients. Outpatients for COPD diseases are most sensitive to air pollution and meteorology factors, followed by allergic rhinitis, asthma, and pneumonia diseases. In the context of sex difference to air pollution and meteorological factors, male outpatients are more sensitive than female outpatients in the 16–65 age groups, while female outpatients are more sensitive than male outpatients in the young 0–15 age groups and in the eldest age groups. In total, female outpatients are more sensitive to air pollution and meteorological factors than male outpatients.

## Introduction

Taiwan contains high density of industrial factories using fossil fuel as a main source of energy to power Taiwan's economy [1]. As such, the air above Taiwan naturally contains heavy emissions from both industrial processes and mobile use every day. The air is polluted [2], [3]. The industrial fruit is explicitly counted as the shining figures of significant gross industrial output every year. However, the air pollutants suspended in the air affect people who live in the county and the outcomes of polluted air on public health remains to be quantified. [4]. The cost of air pollution on human health needs to be counted as well so as to give a more balance picture of short-term fossil-fuel burning economy fruit with respect to the long-term impact on public health [5]. After all, the main motivation for the economy growth is for the long-term and sustainable welfare of the society. What will be the human health cost reflected in these waxing and waning of transient industrial figures?

Happily, Taiwan has a very good National Health Insurance (NHI) program for a high density population of 23 million [6], [7]. The NHI is characterized by good accessibility (Taiwan citizen can see any doctor without referral, and may also go to any levels of hospitals directly), comprehensive coverage (cheap and abundant care), short waiting times, low cost, high coverage rate (99.6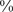 of Taiwan population is covered by NHI in 2010), and a comprehensive and detailed nationwide NHI database that keeps every detailed record of the use of NHI [7], [8]. NHI has contracted 92.1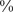 of all hospitals and clinics in Taiwan in 2010 [9].

The NHI database enabled studies of association between air pollution and hospital admissions for cardiovascular disease [6], chronic obstructive pulmonary disease (COPD) [10], asthma [11], lower respiratory tract illness [12], and pneumonia [13]. In these works, NHI database provides quantified hospital admissions for above mentioned diseases, which were then analyzed against ambient air pollution levels obtained from a network of ambient air monitoring stations operated by Taiwan Environmental Protection Administration (EPA) [2]. People often get very ill to get admissions to hospitals. Mortality rate is another measure frequently used to quantify the impact of air pollution on public health [4]. Most of the time people visit hospitals to seek brief consultation and examination on their health. The results of these hospital outpatient visits are kept and comprising a large set of medical service records in NHI computer database [8]. This big data set reflects the status of the health of a population. As such, the use of outpatient data has become an important tool to understand the association between air pollution and public health [14], [15], [16], [17], [18].

Previous studies use NHI database to focus on the association between air pollution and hospital admissions. For example, to associate air pollution and hospital admissions for asthma during the period 1996–2003 [11], and for cardiovascular disease during the period 1997–2001 [6] in Taipei; and to associate air pollution and hospital admissions for pneumonia in Kaohsiung during the period 1996–2004 [13]. A few studies reported association between daily outpatient visits and daily air pollution levels. For example, *Hwang and Chan* [12] used the NHI database to study effects of air pollution on daily clinic visits for lower respiratory tract illness in 50 townships in Taiwan during 1998. In this work we used outpatient data from a hospital in a heavily industrial area in northern Taiwan to study the association between daily outpatient visits and daily air pollution levels in the context of a heavily polluted atmospheric environment during the period 2007–2011.

## Data and Methods

### Ethics Statement

The statistics of daily hospital visits used in this work is openly published each year by the hospital [19], [20], [21], [22]. Only the frequencies of visits for diseases are used in the study. No details of patient's personal information are involved in this work. As such, no ethics committee approving is needed. No written consent from patients, or from the next of kin, caretakers, or guardians on the behalf of minors/children parents are needed.

### 1. Hospital Visit Data

**Figure 1:**
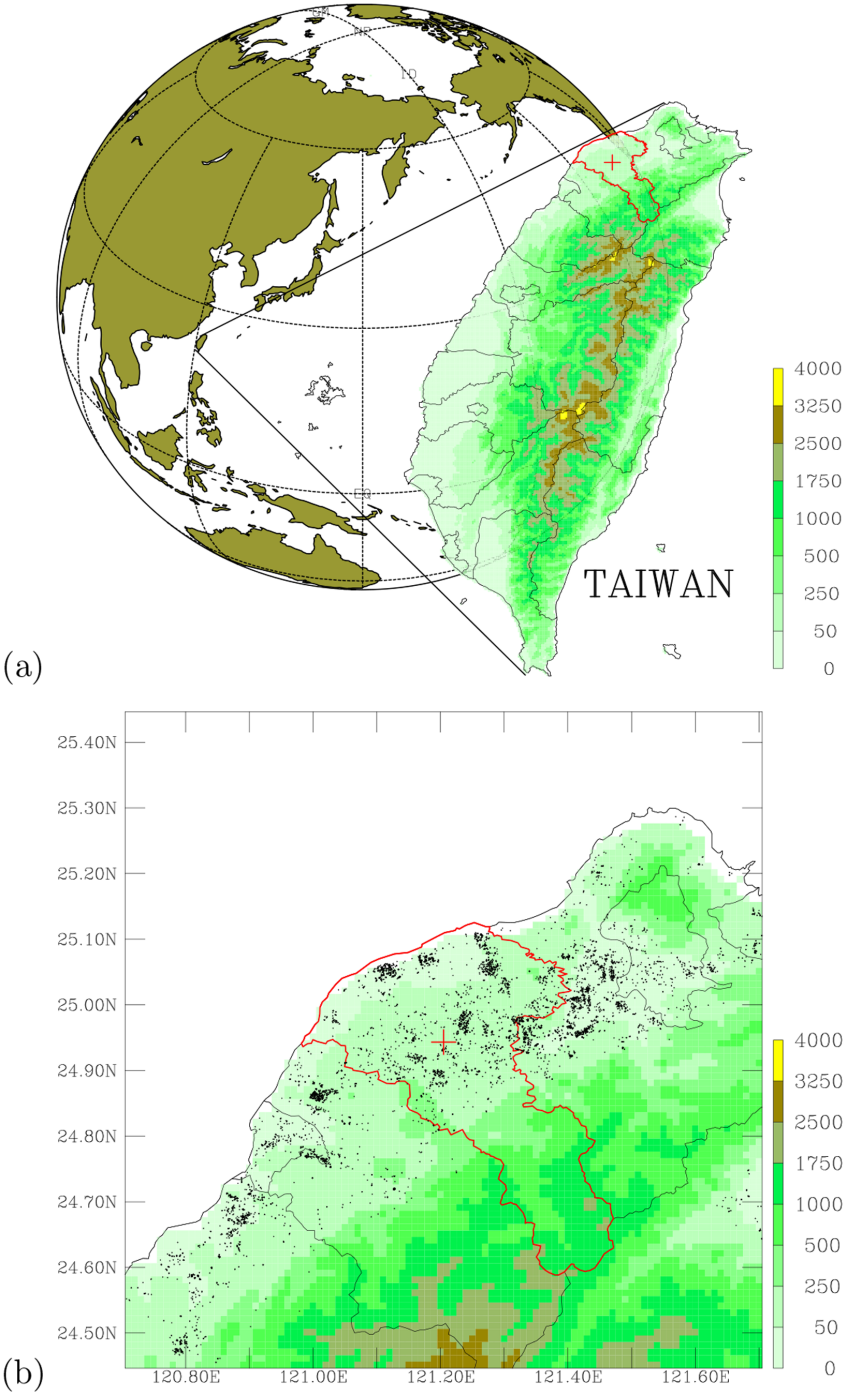
Geographical location of the study area. (a) Location of the Landseed hospital (red cross) in Taiwan's Taoyuan County (area encircled with red solid line). (b) A high resolution map showing topography (shaded colors; in the unit of meter) in the northwestern Taiwan, Taoyuan County (area encircled with red solid line); the Landseed hospital (red cross), and significant industrial emission sources (black dots).

The Landseed hospital (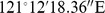, 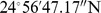) is located in the Taoyuan County, which is in northern Taiwan (Figure 1(a)). Taoyuan County is one of the top major industrial counties in Taiwan, contains several leading industrial figures. Table 1 shows a statistics for Taoyuan county, while the spatial distribution of point industrial emission sources is shown in Figure 1(b). Most of the outpatient visits to the Landseed hospital come from local community in Taoyuan County. About 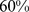 of all visits are from nearby Chung-Li City and Ping-Jeng City, and more than 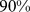 of daily outpatient visits are from Taoyuan County. The demographical nature of outpatients strongly indicates that the hospital is a local community hospital. Given the facts that Taoyuan County contains very heavy industrial production (Figure 1(a) and Table 1) and good records of ambient air monitoring data, the combination of local community outpatient visit data with air pollution monitoring data makes it ideal to look for the association between ambient air pollution and public health.

**Table 1:** Statistics for Tao-Yuan County (TYEPA, 2012; www.tyepb.gov.tw).

Population	2,010,000
Spatial Size	1,220.95 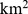
Factories	10,412 (2,718 are under EPA 97 regulation)
Stacks	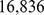
Total Factories in Taiwan	85,752 (Mar 2007; www.moeaidb.gov.tw)
Incinerators	21
Petro Stations	297 (Number 1 in Taiwan)
Industrial Areas	22 (No. 1 in Taiwan; 7 are major industrial areas)
Taiwans Top 500 Major Manufactures	More 1/3 in Tao-Yuan
Display Screen Manufactures	13 (Total 19 in Taiwan)
Industrial Productions	NTD 2.32 Trillions (USD 77 Billions)
	No. 1 in Taiwan; greater than Hsin-Chu Science Park
Mobile Vehicles	1,780,000
Total Mobile Vehicles in Taiwan	20,341,288 (Jan 2007; www.motc.gov.tw)

A list of International Classification of Diseases, 9th Revision (ICD-9), for respiratory system were selected from outpatient visit database for this work. There are four diseases from which data of daily outpatient visits were analyzed and their correlations with daily air pollutants and meteorology were calculated. These diseases are allergic rhinitis (477), asthma (493), pneumonia (480–486), and COPD (490–493).

The hospital contains very detailed records of daily outpatients for each of the disease category. These data extend the period 2007–2011. The daily outpatient data for each disease category are divided into three age groups: ages 0–15, 16–65, and above 66 years old. The divisions of age groups are similar to *Hajat et al.* [23], *Hajat et al.* [24], and *Jalaludin et al.* [18].

Let's denote 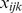 as a one-dimensional vector, representing a time series of daily outpatient numbers for each *i* disease category at each *j* age group and each *k* year. Hence, we can write 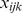 as following,
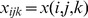
(1)

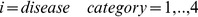
(2)

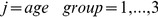
(3)

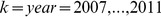
(4)


### 2. Ambient Air Pollution and Meteorological Data

In this work we use ambient air pollution and meteorological data obtained from Chung-Li station (close to the hospital), which is one of the more than 80 ambient air monitoring stations operated by Taiwan EPA [2], [25]. This network of ambient air monitoring stations has started its current scale of operation since 1994. Each station reports hourly measurements of air pollutants and meteorology. A total of 12 observational variables from Chung-Li station are used in this work. These variables contain 7 main air pollutants (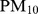, 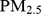, 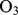, CO, NO, 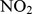, and 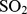) and 5 key meteorological observations (temperature, precipitation, wind direction, wind speed, and relative humidity). Let's denote 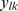 as a vector for these air pollution and meteorological variables. Hence, we can write
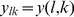
(5)

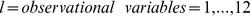
(6)

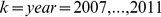
(7)


### 3. Test of Normality for Hospital, Air Pollution, and Meteorological Data

The daily hospital outpatient data is continuous in day, but the hospital does not provide regular outpatient service during weekends (Saturday and Sunday), and other public holidays (Lunar New Year, Moon Festival, Dragon Boat Festival, etc). As such, data that occurred during the public holidays are removed to prevent artificial bias (zero outpatient visits) introduced by these holidays (holiday effect) in the calculation of correlation coefficients. Air pollution and meteorological data during the public holidays are also removed accordingly to avoid holiday effect. We note that if the holiday effect not removed from the time-series data, the correlation coefficients exhibit (not shown here) overwhelmingly associations between the diseases and NO, 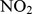, CO, and 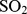.

We then compute observed frequency distribution, which is calculated by binning daily data into 15 bin intervals for each year and for each data. The 15-bin intervals is empirically determined to give sufficient resolution of the data when testing data normality with respect to the continuous normal distribution.

Given an observed frequency distribution one can calculate its mean value and standard deviation. With a mean value and its standard deviation, one can calculate an expected frequency distribution of data if the data is normally distributed [26]. Finally, we can access the normality of observed data by testing the observed frequency distribution against its theoretical normal distribution using statistical methods (e.g. the Chi-square test) following *Press et al.* [26].

### 4. Correlation Coefficient Analysis

Given the outpatient vector 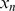 and variable vector 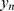, we compute correlation coefficient following *Press et al.* [26]. A measure of significance of this correlation coefficient *r* is obtained by computing the Student's *t* probability *P* following *Press et al.* [26]. The small values of *P* indicates a significant correlation.

In addition, meteorological factors that might play a confounding role are separately considered as warm days (temperature above 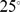
C) and cool days (temperature below 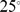
C) in this work, following the method proposed by *Chang et al.* [6], *Cheng et al.* [13], and *Yang et al.* [11]. *Hwang and Chan* [12] also include temperature as a confounding variable in their analysis. Since warm days are often associated with high relative humidity and cool days are often associated with low relative humidity [27], the stratification of days between warm and cool days also separates the effect of relative humidity from high to low.

### 5. Multiple Linear Regression Model Analysis

In this work we use multiple linear regression analysis to account for confounding effects when associating daily hospital visits for diseases with air pollutants and meteorological factors. Multiple linear regression analysis has been widely used to control for confounding factors when predicting dependent variables from independent variables [28], [29], [30], [31], [32], [33], [34].

We have developed multiple linear regression models for association analysis following the methods described in *Kleinbaum et al.* [35], which contains detailed mathematical formulas for the multiple linear regression models, equations to compute statistical significance of regression results, and a rich example data set that demonstrate the results from various multiple linear regression models. *Kleinbaum et al.* [35] use SAS package to carry out multiple linear regression computation. However, in order to facilitate the efficiency of processing large data set from medical and meteorological sources, it is necessary to build computational models from the methods described in *Kleinbaum et al.* [35].

The multiple linear regression models we have developed for this work have been carefully and successfully tested against data from *Kleinbaum et al.* [35]. We have run our models through data in Table 5.1, which shows observations on systolic blood pressure (SBP) and age for a sample of 30 individuals. We have compared our model results with edited SAS output for Table 5.1 in page 52 of *Kleinbaum et al.* [35]. We have also run our model through data shown in Table 8.1, containing a list of weight, height, and age of a random sample of 12 nutritionally deficient children. Our models were compared with a list of edited SAS output from 6 multiple regression models (shown in pages 124–126). Our detailed comparisons show consistent results between SAS calculations and our model calculations. These comparisons demonstrate our models are accurate when compared with the SAS package. We then use these multiple models to run through the medical and the meteorological data.

Table 2 shows a typical example of our multiple linear regression analysis. A list of 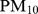-based multiple linear regression models that were systematically used to run the medical and meteorological data. The purpose is to find the best model that associate air pollutant 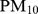 with outpatient visits for each of respiratory diseases discussed in this work. Following *Kleinbaum et al.* [35], we use 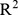 and statistical significance level (*P* value) to select the best model for our analysis.

**Table 2:** List of 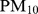-based Multiple Linear Regression Models.

Model1	OP =	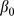 + 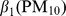
Model2	OP =	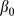 + 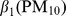 + 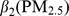
Model3	OP =	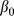 + 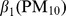 + 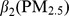 + 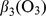
Model4	OP =	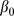 + 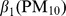 + 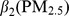 + 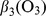 + 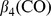
Model5	OP =	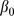 + 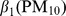 + 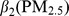 + 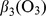 + 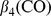 + 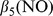
Model6	OP =	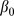 + 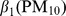 + 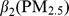 + 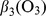 + 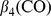 + 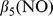 + 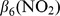
Model7	OP =	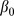 + 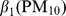 + 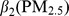 + 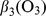 + 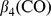 + 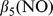 + 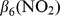 + 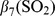
Model8	OP =	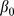 + 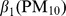 + 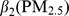 + 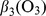 + 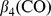 + 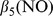 + 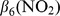 + 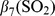 + 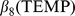
Model9	OP =	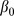 + 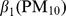 + 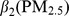 + 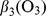 + 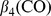 + 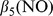 + 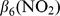 + 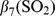 + 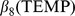 + 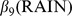
Model10	OP =	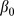 + 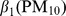 + 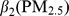 + 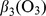 + 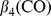 + 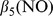 + 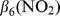 + 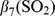 + 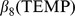 + 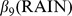 + 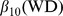
Model11	OP =	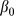 + 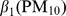 + 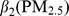 + 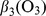 + 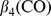 + 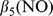 + 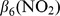 + 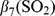 + 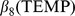 + 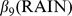 + 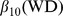 + 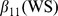
Model12	OP =	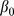 + 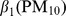 + 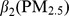 + 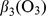 + 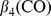 + 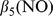 + 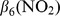 + 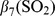 + 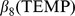 + 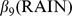 + 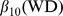 + 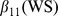 + 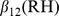

OP: outpatient visits; TEMP: temperature; RAIN: rainfall; WD: wind direction; WS: wind speed; RH: relative humidity.

Table 2 shows a list of 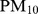-based model analysis. Similarly, we have systematically run twelve multiple linear regression models based on 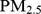, 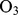, CO, NO, 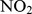, 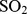, temperature, rainfall, wind direction, wind speed, and relative humidity, respectively. As such, a total of 144 models were run for each of respiratory disease, in each year of 2007–2011, considering male and female outpatients separately, and for each of three age groups of outpatients (0–15, 16–65, and above 66 years). Hence, we have completed a total of 17,280 model analysis (4 respiratory diseases 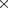
144 models/disease 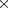
5 years 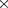
2 sex groups/year/age group 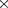
3 age groups/sex). The results that are statistically most significant are shown in the following section.

## Results

### 1. Daily Outpatients For Respiratory System Diseases During 2007–2011

**Figure 2:**
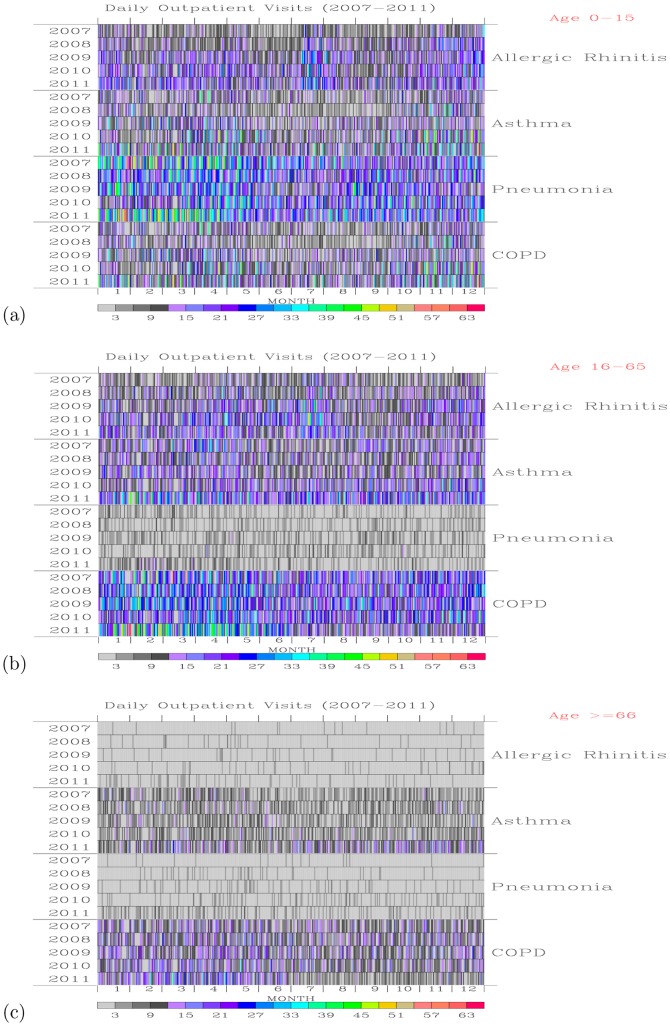
Daily outpatient visits. For the ages (a) 0–15, (b) 16–65, and (c) above 66 year-old group of people for each of the respiratory system diseases during the 2007–2011 period.

Figure 2 shows daily outpatient visits for the 0–15, 16–65, and above 66 year-old age group of people for each of the respiratory system diseases during the 2007–2011 period. For the age 0–15 group of outpatients (Figure 2a), most of the daily visits were for pneumonia disease, followed by allergic rhinitis, asthma, and COPD. Peak visits for pneumonia disease during January–April, while the period with low pneumonia outpatients occurred during summer months of June and July. For allergic rhinitis disease, more daily hospital visits occurred during late February to early May, July–August, and November–December than other months. The first and the last periods are normally associated with poor air quality [36], while the summer months of July August may be associated with high relative humidity.

For the 16–65 age group outpatients (Figure 2b), most of the hospital visits were for COPD disease, followed by asthma and allergic rhinitis. Few visits were for pneumonia. For COPD outpatients, more visits occurred during winter to spring months than the summer months. For the group of outpatients with ages above 66, their visits are mainly associated with COPD and asthma (Figure 2c). Table 3 gives a summary of the total number of outpatient visits for each disease of the respiratory system during 2007–2011.

**Table 3:** List of Total Outpatient Visits According to Disease and Year.

Disease	2007	2008	2009	2010	2011
Allergic Rhinitis	5118	6803	8413	9041	9021
Asthma	7779	7475	7910	8988	12458
Pneumonia	7878	7036	7066	6989	9051
Chronic obstructive pulmonary disease	11061	11225	12159	12082	13076

### 2. Air Pollution and Meteorology During 2007–2011

**Figure 3:**
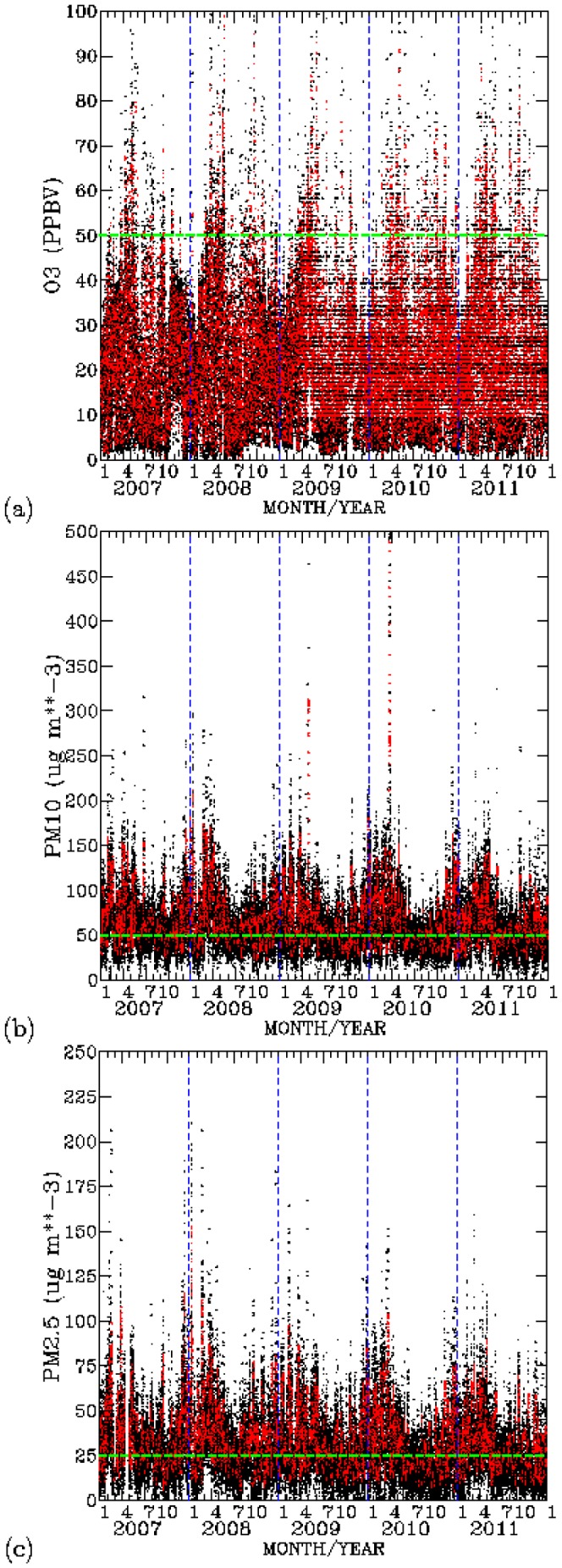
Time-series plots of air pollutants. (a) 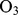 (ppbv), (b) 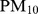 (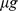 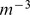), and (c) 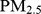 (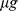 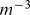) for the 2007–2011 period. Black dots indicate hourly data, while red dots indicate 8-hourly mean for 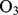, and 24-hourly mean for 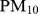 and 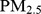. Green dashed are pollutant levels according to the WHO guidelines.

Time-series plots of hourly ambient air monitoring of 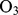, 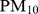, and 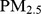 at EPA Chung-Li station for the period 2007–2011 are shown in Figure 3. Also shown in the figure are the 8-hourly mean 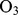, 24-hourly mean 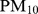, and 24-hourly mean 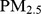. Elevated levels of hourly and 8-hourly mean 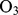 mostly occurred during spring and summer seasons, Figure 3(a). Around 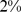 (2007), 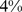 (2008), 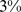 (2009), 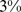 (2010), and 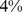 (2011) of 8-hourly mean 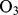 are higher than the World Health Organization (WHO) air quality guidelines for 8-hourly mean 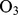 [37].

For 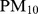, Figure 3(b), both hourly and 24-hourly mean levels are mostly above the 24-hourly mean levels of 50 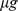 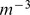 recommended by WHO air quality guidelines for 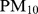. Around 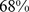 (2007), 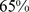 (2008), 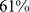 (2009), 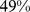 (2010), and 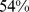 (2011) of 24-hourly 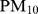 are higher than the WHO standard for 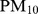. For 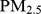, around 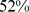 (2007), 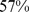 (2008), 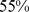 (2009), 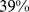 (2010), and 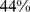 (2011) of 24-hourly mean levels are are above the 24-hourly mean levels of 25 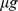 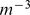 recommended by the WHO standard for 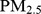. Elevated levels of 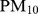 and 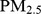 frequently occurred during the winter, spring, and fall seasons. Summer is the only season when ambient levels of 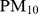 and 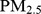 are low. Taiwan is under the strong influence of the southerly Asian summer monsoon flow in the summer season. This monsoon flow brings clean tropical air to the entire county, resulting in a much reduced ambient air pollution in summer [36].

**Figure 4:**
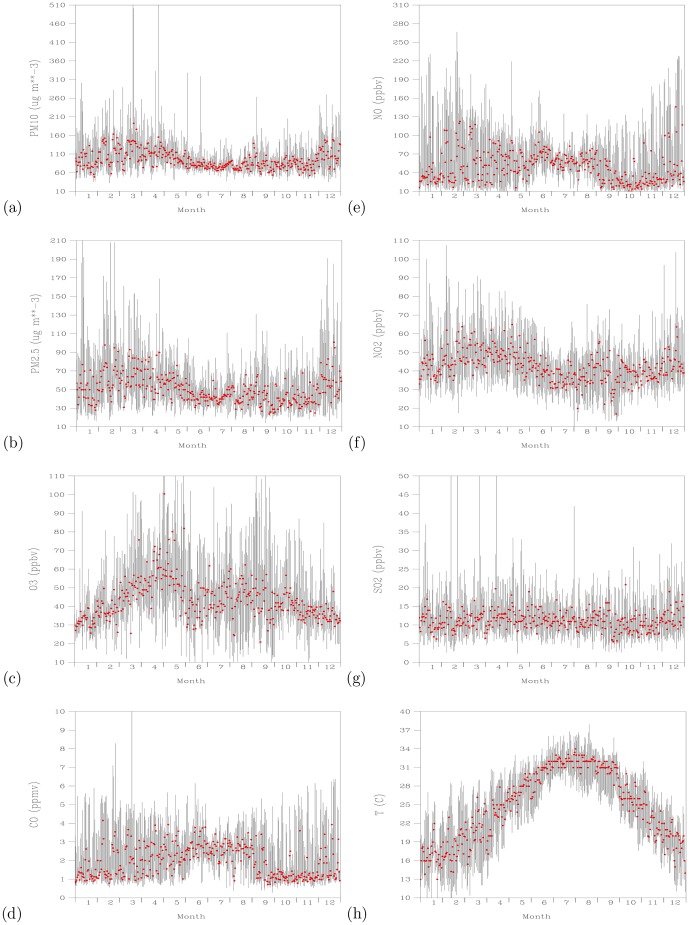
Time-series plots of daily maximum (black lines) for the same Julian day of 2007–2011 period. For (a) 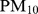 (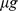 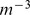), (b) 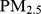 (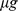 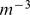), (c) 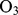 (ppbv), (d) CO (ppmv), (e) NO (ppbv), (f) 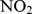 (ppbv), (g) 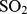 (ppbv), and (h) temperature (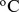). Red dots indicate medium values for each Julian day.

Figure 4 shows time-series plots of daily maximum values of 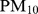, 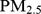, 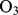, CO, NO, 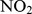, 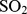, and temperature for the 2007–2011 period. Range of the daily maximum values for each variable during 2007–2011 are shown as vertical lines. Hence, the longer the vertical lines, the larger the variations that had occurred in that day of the year. Daily maximum levels of 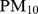 greater than 50 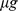 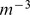 (Figure 4(a)) and 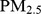 greater than 25 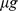 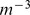 (Figure 4(b)) can overwhelmingly occur each day of the period 2007–2011. Daily maximum levels of 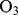 higher than 50 ppbv mostly occurred during March–May and July–October months (Figure 4(c)). Except for the June–August months, daily maximum levels of CO (Figure 4(d)) and NO (Figure 4(e)) exhibit large variability. Daily maximum levels of 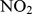 show clear seasonal cycle (Figure 4(f)). High levels of 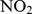 occurred during the winter to spring seasons, followed by low levels of 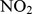 in summer season. 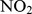 levels rise again in the fall to winter seasons. Daily maximum levels of 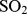 show less clear seasonal cycle than 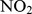 (Figure 4(g)). The medium values of daily maximum 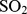 are centered about 10 ppbv. Large variabilities of daily maximum 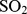 close to 20 ppbv frequently occurred all year round. Daily maximum temperatures follow the marching of the season, with high temperatures occurred during July–August and low temperatures occurred during January–February (Figure 4(f)). Temperatures in summer months show less variability than those occurred in the winter to spring months.

The observations show that the variations of daily maxima for CO and NO are smaller and the minimum concentrations for both species are higher during June–August months than during the rest of other months. Since the dominating flows during the June–August months are coming from the south directions [36], the persistently high minimum CO and NO levels indicate sustained contribution from local pollution sources. The ability to reach low CO and NO during the rest of other months indicate the effect of large-scale flows from the north directions to bring daily maximum CO and NO to low concentration.

### 3. Test of Normality for Air Pollutant and Meteorology Data

**Figure 5:**
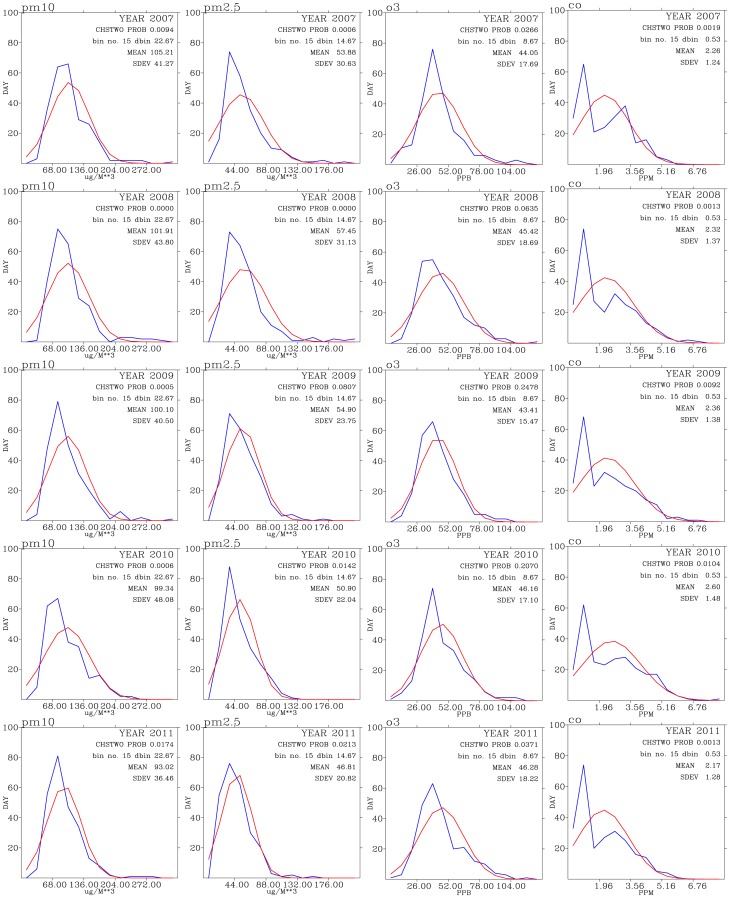
Test of normal distribution for 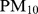 (left panels), 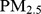 (second from the left panels), 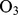 (second from the right), and CO (right panels) for the years from 2007 (top row) to 2011 (bottom row).

**Figure 6:**
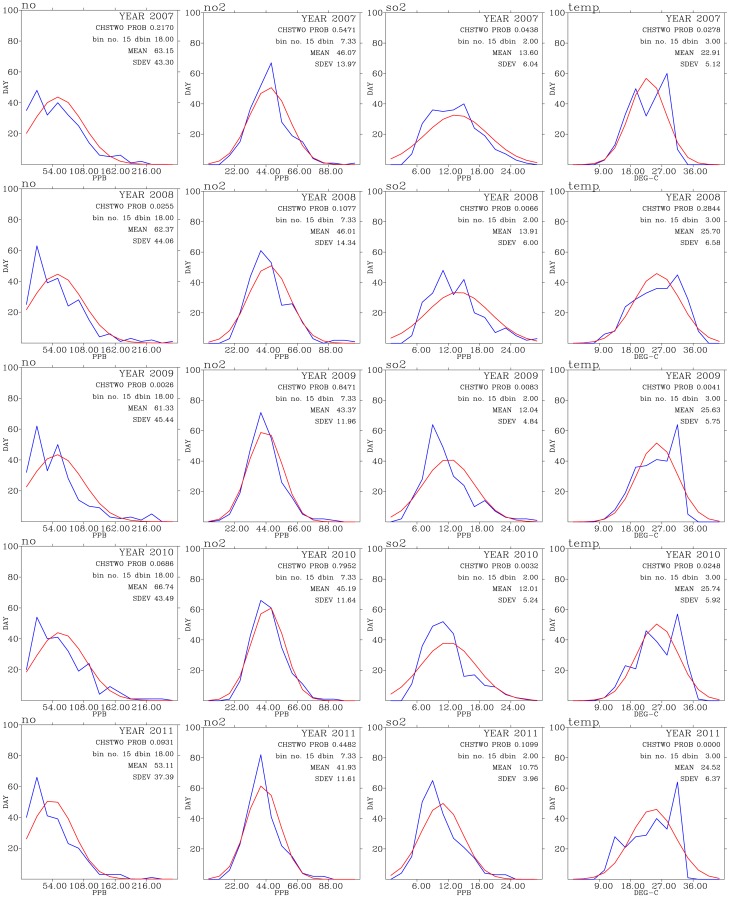
Test of normal distribution for NO (left panels), NO2 (second from the left panels), SO2 (second from the right panels), and temperature (right panels) for years from 2007 (top row) to 2011 (bottom row).

**Figure 7:**
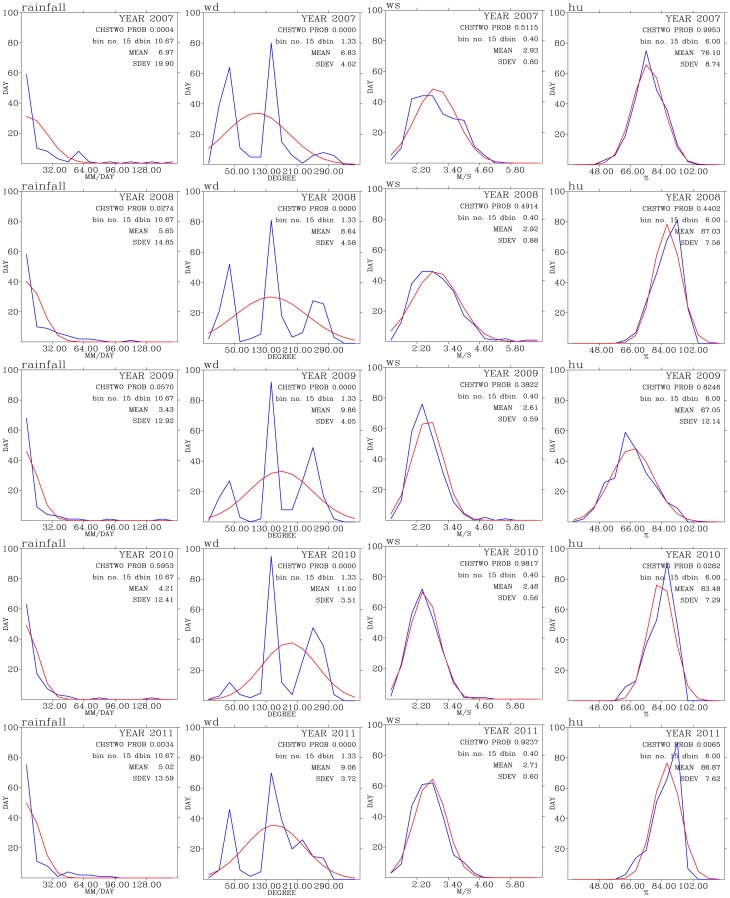
Test of normal distribution for rainfall (left panels), wind direction (second from the left panels), wind speed (second from the right panels) and relative humidity (right panels) for years from 2007 (top row) to 2011 (bottom row).

Figure 5, Figure 6, and Figure 7 shows test of normality for air pollutants and meteorological data for each year of the analysis period of this work. Except for CO, rainfall, and wind direction, the rest of the data exhibit patterns of normal distribution when binned data are compared with the expected normal distribution.

The mean values for 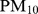 for these five years have decreased from 105 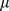
g 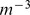 in 2007 to 93 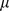
g 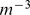 in 2011. However, stadard deviations of 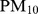 varies between 36 and 41 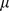
g 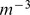. This indicates that daily maximum 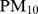 in most days of each year are much higher that those WHO guidelines. This is consistent with the time-series analysis shown in Figure 3(b). This result indicates that Taoyuan area frequently under the influence of processes (local emissions and/or non-local sources) that persistently sustain elevated levels of 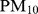.

Means of 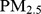 levels decrease from 53–57 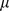
g 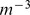 during 2007–2008 to 46 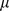
g 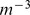 in 2011. The standard deviations for 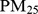 has also decreased from around 30 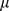
g 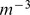 in 2007 to 20 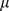
g 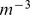 in 2011. This indicates that about 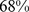 of days in 2007 show daily maximum 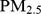 varies between 26 and 66 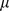
g 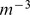 [50]. Most of the days in Taoyuan are having levels of 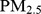 above WHO guidelines (see also Figure 3(c)). The mean values for NO, 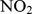, and 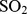 all show decreasing trends during the 2007–2011 period (Figure 6), while the mean values for 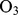 has slightly increased, from 44 ppbv in 2007 to 46 ppbv in 2011.

These analyses consistently show signs of decrease in primary pollutants while slightly increase in the secondary pollutant 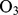 in the air. This could indicate the reduction in local industrial activities either by their relocation to other countries or by the effectiveness of more stringent emission control policies.

The wind directions show three modes of peak frequency distribution centered around 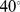, 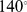, and 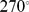 directions. The highest frequency distribution of wind direction come from around 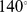 directions, indicating southerly winds. This is an indication of winds mostly occur during summer monsoon season when winds were from the south directions. The wind directions centered around 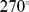 representing westerly winds, which normally occur during the day-time period when the sea breeze prevailed over the Taoyuan area (Figure 1(b)). The winds coming from around the 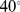 directions representing the prevailing northerly winds which occur mostly during the late fall, winter, and early spring seasons.

### 4. Attributing Disease's Environmental Effect: Test of Outpatient Normal Distribution and Correlation Coefficient Analysis

#### 4.1. Allergic Rhinitis

**Figure 8:**
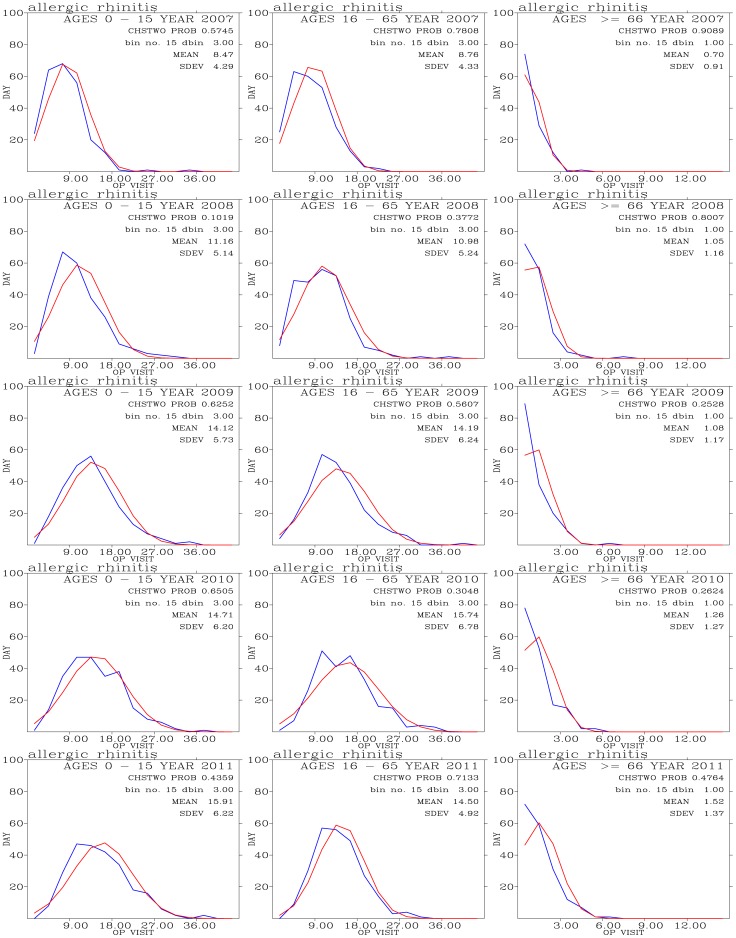
Binned daily outpatient visits for allergic rhinitis (blues curves) and expected normal distribution (red curves) from years 2007 (top row) to 2011 (bottom row); and for outpatients with ages 0–15 (left panels), 16–65 (middle panels), and above 66 (right panels).

Figure 8 shows frequency distribution of outpatient visits for allergic rhinitis and comparison with expected normal distribution. Outpatients for this disease concentrate on people with ages 0–15 and 16–65. The mean values of outpatient visits shows slightly increase trend during the 5-year period. The outpatient visits shows close resemblance to the expected frequency distribution from normal distribution.

**Figure 9:**
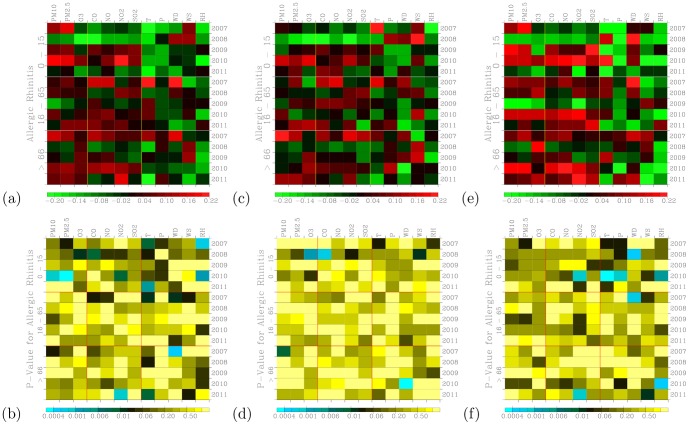
Distribution of correlation coefficients when correlating hospital visits for allergic rhinitis with air pollutants and meteorology for three age groups during (a) the 2007–2011 periods. (c) The same as in (a) but to control for warm days (temperature above 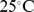); and (e) to control for cold days (temperature below 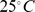). Significance of correlation are shown in (b), (d), and (f).

Figure 9(a) shows distribution of correlation coefficients when correlating hospital visits for allergic rhinitis with air pollutants and meteorology for three age groups during the 2007–2011 periods. Figure 9(b) shows a measure of significance for the correlation coefficients calculated in Figure 9(a). The small values in Figure 9(b) indicate significant correlation [26].

The 0–15 age group of outpatients for allergic rhinitis are mostly and positively correlated with PM_10_ and PM_2.5_. Temperature shows significantly negative correlations for this young age group of outpatients. Temperatures do not exhibit a clear association with both the 16–65 and above 66 age groups of outpatients. Air pollutants such as PM_10_, PM_2.5_ O_3_, CO, NO, NO_2_, and SO_2_ tend to show positive correlations with visits for allergic rhinitis when compared with meteorological factors. These pollutants have been shown to be related to allergic rhinitis [17].

When controlled for temperature effect, both cool (Figure 9(c)) and warm (Figure 9(e)) days also exhibit positive correlation coefficients. However, the correlation coefficients during cool days are more pronounced and higher than those during warm days. Also the significance of correlation occurred during the cool days (Figure 9(f)) are stronger than those during the warm days (Figure 9(d)). This could indicate low temperatures can act to acerbate the symptoms allergic rhinitis. Distribution of correlation coefficients during cool days (Figure 9(e)) are more pronounced than the effects of temperature were not controlled (Figure 9(a)). Hence, there is a confounding effect of temperature when correlating daily outpatient visits for allergic rhinitis with air pollution levels.

*Hajat et al*. [23] found strong association between 4-day lag SO_2_ and O_3_ measurements and the number of consultations for allergic rhinitis in London. PM_10_ and PM_2.5_ are less significant in associating with the allergic rhinitis following *Hajat et al*. [2001]. On the other hand, *Wong et al*. [38] found significant and positive association between PM_10_ and upper respiratory tract infections, while *Wong et al*. [2006] showed significant association between first visits for upper respiratory tract infections and an increase in the concentrations of NO_2_, O_3_, PM_10_, and PM_2.5_ in Hong Kong.

We note that positive correlations may not exit through all 5 years. For example, allergic rhinitis with PM_10_ in 2008 are negative while the rest of other four years are positive for the age 0–15 group of outpatients (Figure 9(a)). The majority of the correlation coefficients are positive.

#### 4.2. Asthma

**Figure 10:**
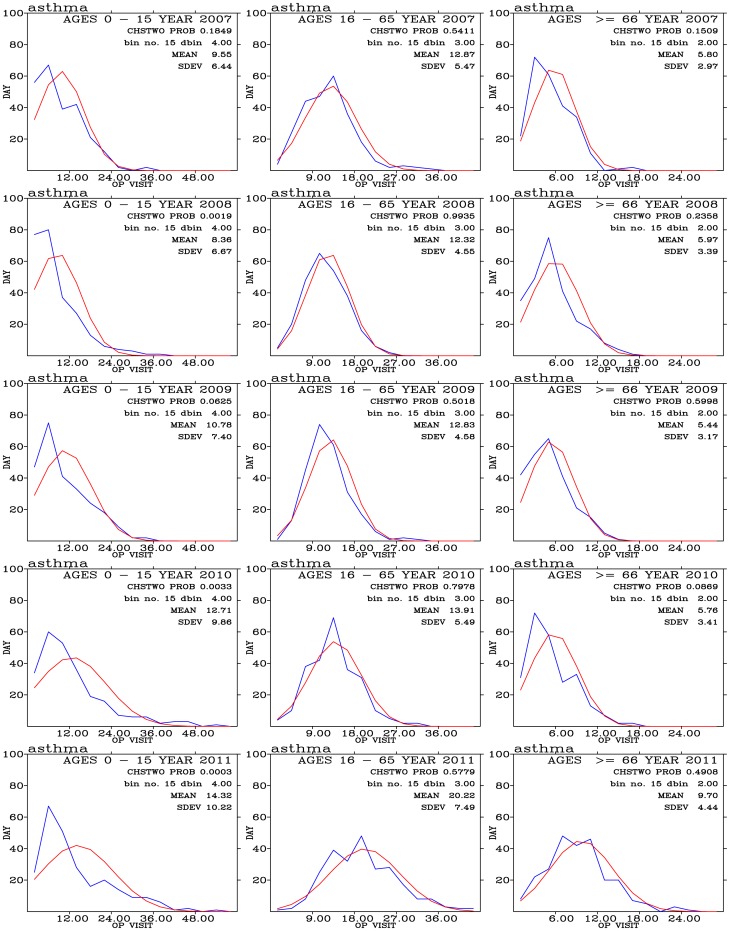
Binned daily outpatient visits for asthma (blues curves) and expected normal distribution (red curves) from years 2007 (top row) to 2011 (bottom row); and for outpatients with ages 0–15 (left panels), 16–65 (middle panels), and above 66 (right panels).

Frequency distribution of daily outpatient visits for asthma is shown in Figure 10. These daily outpatient distribution shows remarkably close resemblance to the expected normal distribution in ages 16–65 and above 66 groups of people. The largest group of outpatients is from the 16–65 age group of people. Significant increases in the mean values of daily outpatient visits are seen in people with ages 16–65 and above 66.

**Figure 11:**
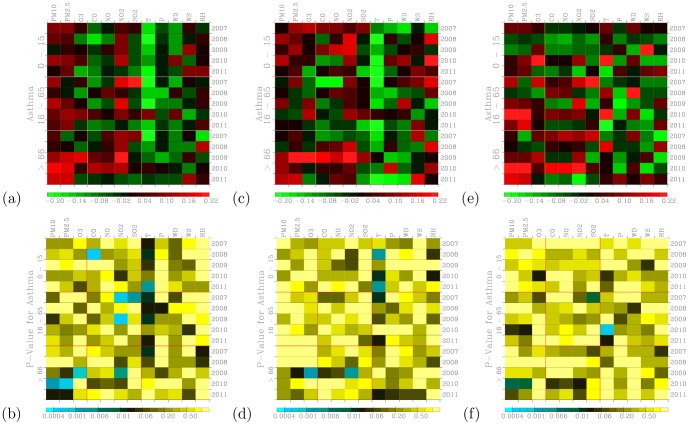
Distribution of correlation coefficients when correlating hospital visits for asthma with air pollutants and meteorology for three age groups during (a) the 2007–2011 periods. (c) The same as in (a) but to control for warm days (temperature above 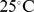); and (e) to control for cold days (temperature below 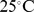). Significance of correlation are shown in (b), (d), and (f).

Air pollutants such as PM_10_, PM_2.5_, and NO_2_ exhibit positive correlations with the daily outpatient visits for asthma (Figure 11(a)). These correlations are very significant (Figure 11(b)), especially for outpatients with age above 66 years old. Our findings of strong and significant correlations between asthma and PM_10_ and PM_2.5_ are consistent with *Schwartz et al*. [39] and *Sheppard et al*. [40]. Both studies showed the daily counts of emergency room visits persons under age 65 and non-elderly were significantly associated with PM_10_ on the previous day in Seattle. *Sunyer et al*. [41] found daily admissions for asthma in adults increased significantly with increasing ambient levels of NO_2_ in four European cities. *McConnell et al*. [42] found positive associations between air pollutants (PM_10_ and NO_2_) and bronchitic symptoms in Southern California children with asthma. On the other hand, decrease in particulate air pollution were associated with decrease in asthma and bronchitis admissions of children [43] and mortality rates [44].

Outpatients for asthma disease negatively correlated with temperature and these correlations are significant. This result is consistent with *Whittemore and Korn* [45], who showed more asthma attacks on cool days in the Los Angeles area. Asthma outpatients also show negative correlations with wind direction. This indicates westerly winds tend to have lower asthma outpatients, while easterly winds tend to have higher number of asthma outpatients. Easterly winds are associated with winds from the ocean directions, while easterly winds are associated with winds from inland directions. More industrial factories and transportation vehicles are located from inland directions than from ocean directions.

For asthma, age 0–15 group of outpatients shows more prevailing positive correlations with PM_10_, PM_2.5_, O_3_, CO, NO, and NO_2_ during warm days (Figure 11(c)) than during cold days (Figure 11(e)) when positive correlations are mostly associated with PM_10_, PM_2.5_, and O_3_. On the other hand, age 16–65 group of outpatients show pronounced positive correlations with all pollutants during cool days than during days. For outpatients above age 65, occurrence of positive correlation coefficients during both warm and cold cool days are similar.

These results indicate that temperature can play a delicate effect on asthma outpatients. Cool days appear to reduce the effect of air pollutants on asthma symptoms for age 0–15 of outpatients, while effect of air pollutants enhanced during warm days. For older outpatients, age 16–65, effect of air pollutants on asthma symptoms are all enhanced during cool days than during warm days. For the eldest outpatients, age about 66, temperature effects are less clear when comparing cool with warm days. The appearance of highly correlated coefficients also exhibit high levels of significance, Figures 11(d) and 11(f). These results also indicate the confounding effects of temperatures on the association of daily asthma outpatients with air pollution levels.

#### 4.3. Pneumonia

**Figure 12:**
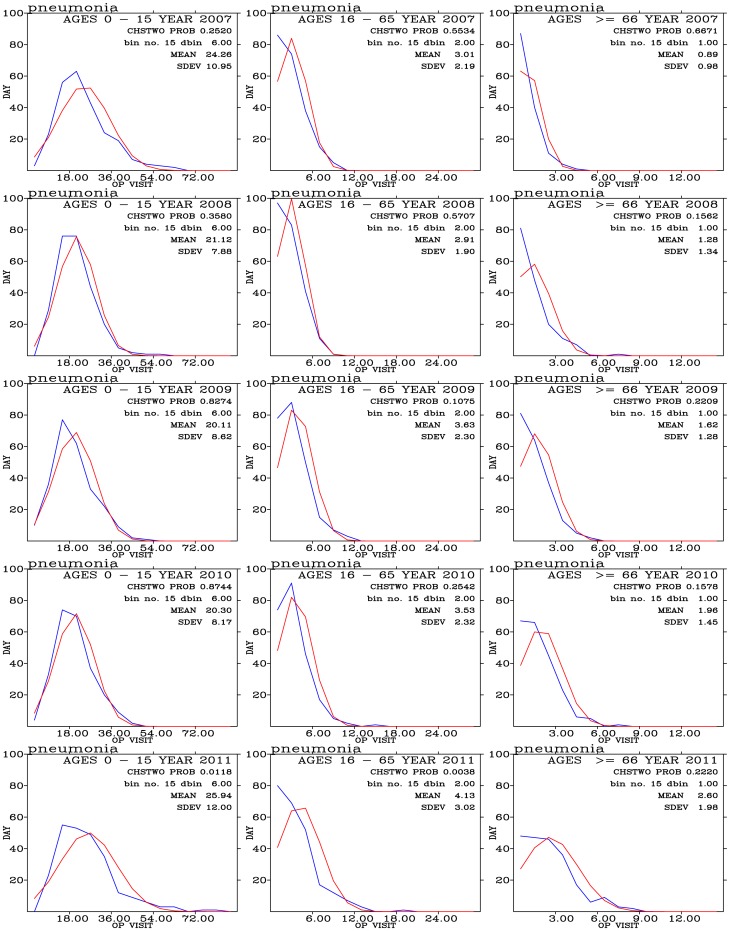
Binned daily outpatient visits for pneumonia (blues curves) and expected normal distribution (red curves) from years 2007 (top row) to 2011 (bottom row); and for outpatients with ages 0–15 (left panels), 16–65 (middle panels), and above 66 (right panels).

Frequency distribution of outpatients for pneumonia disease are shown in Figure 12 for three age group of people during the 2007–2011 period. Most outpatients for pneumonia concentrate on the 0–15 year-old people. The frequency distribution of these outpatients exhibit a good agreement with the expected normal distribution.

**Figure 13:**
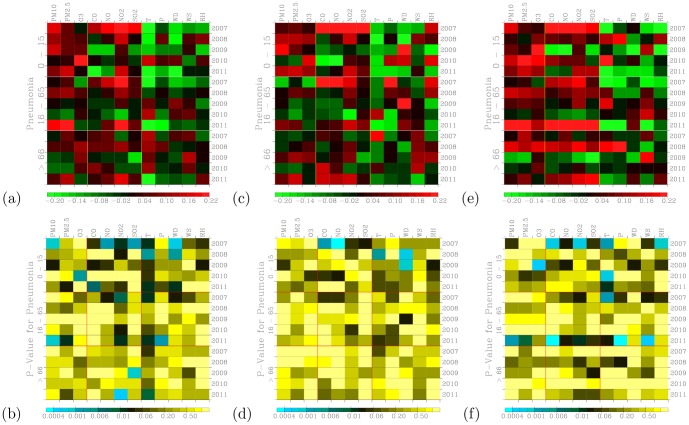
Distribution of correlation coefficients when correlating hospital visits for pneumonia with air pollutants and meteorology for three age groups during (a) the 2007–2011 periods. (c) The same as in (a) but to control for warm days (temperature above 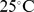); and (e) to control for cold days (temperature below 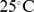). Significance of correlation are shown in (b), (d), and (f).

Pneumonia outpatients are positively correlated with PM_10_, PM_2.5_, O_3_, and NO_2_ for age 0–15 group of outpatients (Figure 13(a)). High values of the these positive correlation coefficients often associated with low P-values (Figure 13(b)), indicating that the correlations are significant. These results are consistent with previous findings. For example, *Penna and Duchiade* [46] found a statistically significant association between the average annual levels of particulates and infant mortality from pneumonia. *Schwartz* [47] found causal association between the increasing particulate air pollution and increase in mortality rates from COPD and pneumonia. In a study of air pollution and infant mortality in Mexico City, *Loomis et al*. [48] showed that NO_2_ and O_3_ are also associated with the infant mortality, but not as consistently as with particulate air pollution.

For pneumonia, cool days (Figure 13(e)) generally exhibit a strong effect on the distribution of air pollutant correlated coefficients than during warm days (Figure 13(c)) and during all days (Figure 13(a)). For the youngest pneumonia outpatients (age 0–15), effects of PM_10_, PM_2.5_, and O_3_ show strongly positive correlation coefficients. Effects of CO, NO, NO_2_, and SO_2_ correlated coefficients are higher during the cool days than during the warm days. These correlations are significant (Figures 13(d) and 13(f)) and similar during cool and warm days. For the older outpatients (ages 16–65, and above 66), effects of air pollutants are significantly enhanced during the cool days than during the warm days.

Our results show the confounding effects of temperature are quite complicated. Effects of PM_10_, PM_2.5_, and O_3_ on pneumonia outpatients are less sensitive to temperature for the youngest group of people but sensitive to other two older groups. Effects of CO, NO, NO_2_, and SO_2_ are all sensitive to temperatures for all three age groups of outpatients.

#### 4.4. COPD

**Figure 14:**
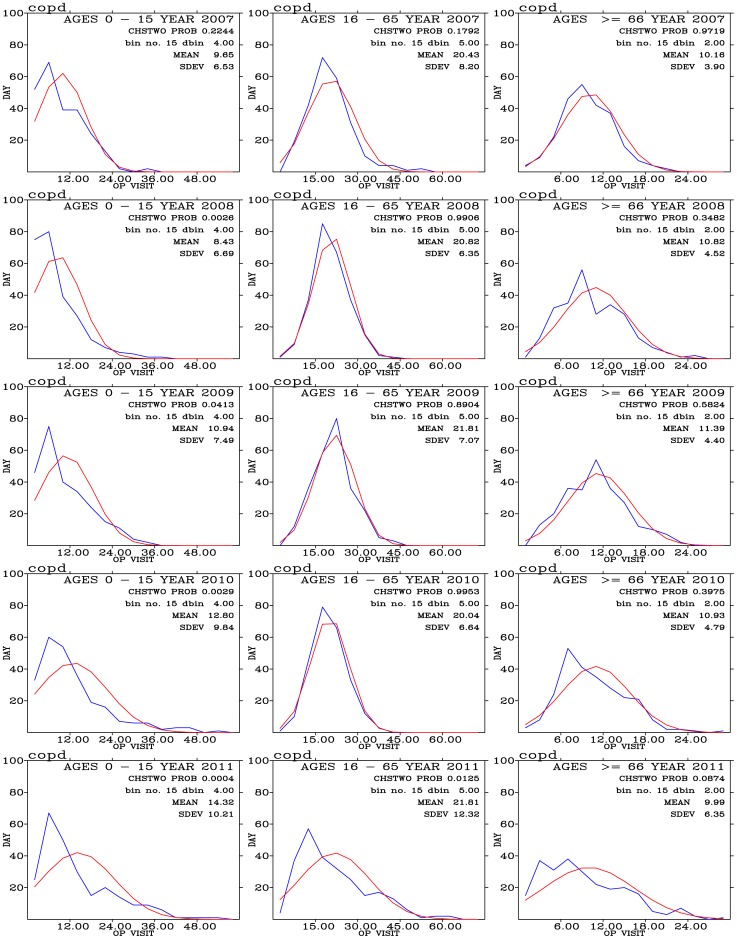
Binned daily outpatient visits for COPD (blues curves) and expected normal distribution (red curves) from years 2007 (top row) to 2011 (bottom row); and for outpatients with ages 0–15 (left panels), 16–65 (middle panels), and above 66 (right panels).

Figure 14 shows frequency distribution of outpatient visits for COPD. The observed frequency distribution of outpatient visits are close to theoretical normal distribution for ages 16–65 and above 66 group of outpatients.

**Figure 15:**
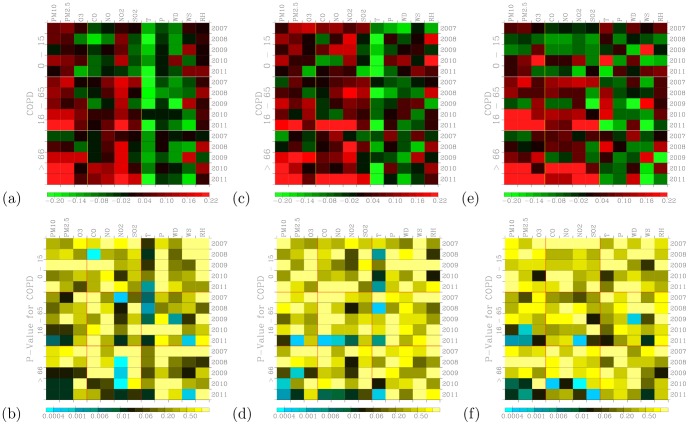
Distribution of correlation coefficients when correlating hospital visits for (a) COPD with air pollutants and meteorology for three age groups during the 2007–2011 periods. (c) The same as in (a) but to control for warm days (temperature above 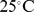); and (e) to control for cold days (temperature below 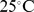). Significance of correlation are shown in (b), (d), and (f).

Outpatient visits for COPD shows overwhelmingly negative correlation with temperature (Figure 15(a)), and these correlations are very significant for thee age groups of outpatients. Positive correlations are seen for PM_10_, PM_2.5_, and NO_2_. These correlations are very significant (Figure 15(b)). Positive correlations also seen for O_3_ and NO but are not as persistently presented as for particulate air pollution and NO_2_. Previous works have shown positive association between particulate air pollution and COPD [47], [49].

For COPD, the youngest outpatients (age 0–15) are more correlated with air pollutants during the warm days (Figure 15(c)) than during the cool days (Figure 15(e)). However, for the other two older group of outpatients (ages 16–65, and above 66), both warm days and cool days show similar distribution of correlation coefficients. COPD are mostly correlated with air pollutants for the oldest outpatients (above 66) and these correlations are significant (Figures 15(d) and 15(f)).

### 5. Multiple Linear Regression Model Analysis

As shown in Table 2, a list of twelve multiple linear regression models were used to find best association between outpatient visits and air pollutants and meteorological factors. Table 4 shows a list of typical results when 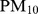-based models were run one by one through the 0–15 age group of male outpatients for allergic rhinitis disease in 2010. These results show that complex models produces statistically significant results (as seen from *F* value, 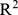, and *P* value) compared with simple models. Regression results are similar when 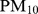, 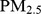, and 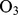 are three major independent variables. Inclusion of CO as an additional independent model variable increase 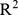, followed by inclusion of another independent model variable 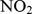. The most significant increase in 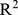 occurs when temperature is included as an independent variable in the model. Addition of relative humidity, rainfall, and winds also increase 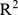 and the statistical significance of our model analysis. Similar results consistently occur when run multiple linear regression models were run through other disease, age group of outpatients, air pollutants, and meteorological factors. These multiple linear regression results are also consistent with the confounding effects of temperature from correlation coefficient analysis shown in the previous section.

**Table 4:** List of 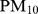-based Regression Coefficients, *F* value, 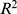, and *P* Value.

Sex	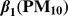	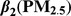	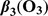	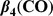	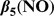	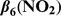	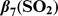	
	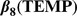	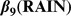	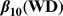	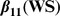	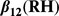	F-value	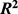	P-value
*Age Group 0–15 Years: Allergic Rhinitis; 2010; Male Outpatients*								
Model1	0.0051					1.98		
							0.01	0.1608
Model2	0.0050	−0.0007				0.99		
							0.01	0.3736
Model3	0.0052	−0.0014	0.0054			0.77		
							0.01	0.5110
Model4	0.0037	−0.0049	0.0031	0.2948		1.73		
							0.03	0.1433
Model5	0.0035	−0.0042	0.0025	0.3464	0.0017	1.39		
							0.03	0.2283
Model6	0.0053	−0.0011	0.0056	0.3707	0.0015	−0.0299		
						1.78	0.04	0.1028
Model7	0.0052	0.0003	0.0059	0.3914	0.0012	−0.0291	−0.0153	
						1.56	0.04	0.1482
Model8	0.0037	0.0084	−0.0038	−0.0956	0.0051	−0.032	−0.0282	
	0.1385					3.01	0.09	0.0031
Model9	0.0032	0.0081	0.0046	−0.1138	0.0054	0.002	−0.0322	
	0.1402	−0.0076				2.78	0.10	0.0040
Model10	0.0027	0.0065	−0.0044	−0.1044	0.0043	−0.0008	−0.0269	
	0.0984	−0.0093	0.834			2.83	0.11	0.0024
Model11	0.0034	0.0054	−0.0045	−0.1462	0.0045	−0.0064	−0.0292	
	0.0947	−0.0088	0.0752	−0.2501		2.67	0.11	0.0030
Model12	0.0019	0.0071	−0.0121	−0.1463	0.0034	−0.0052	−0.0367	
	0.1029	−0.0014	0.0760	−0.3452	−0.0428	2.83	0.13	0.0012

Table 5 shows multiple linear regression analysis of daily outpatients for allergic rhinitis. Our 12-parameter multiple linear regression models shows that the 0–15 age group of male outpatient visits are statistically significant in the 2007 (*P* = 0.0014), 2008 (*P* = 0.0001), and 2010 (*P* = 0.0046) data. NO is the main pollutant and wind speeds is the main meteorological factor that are persistently and positively associated with the increase of outpatient visits. Relative humidity is persistently and negatively associated with the outpatient visits. Low relative humidity predicts higher outpatient visits than high relative humidity. For the female outpatients, only the 2010 data showing statistically significant association (*P* = 0.0002). Positive associations occur with 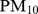, 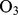, NO, 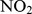, and wind speeds. Negative associations occur with temperature and relative humidity.

**Table 5:** Multiple Linear Regression Analysis of Allergic Rhinitis Outpatient Visits: Regression Coefficients, *F* value, 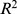, and *P* Value.

Sex	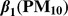	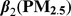	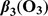	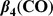	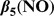	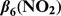	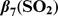	
	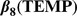	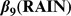	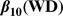	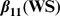	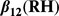	*F*-value	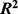	*P*-value
*Age Group 0–15 Years: Allergic Rhinitis*								
2007								
male	−0.0000	0.0144	−0.0039	−0.3585	0.0061	−0.0109	−0.0480	
	−0.1231	−0.0201	0.0545	0.0843	−0.0396	2.97	0.13	0.0014
2008								
Male	−0.009	0.0047	−0.0362	−0.9378	0.0175	0.0215	0.0586	
	−0.0535	0.0088	0.1703	0.0081	−0.0830	3.63	0.15	0.0001
2010								
Male	0.0061	−0.0008	−0.0043	−1.1157	0.0225	0.1039	−0.0532	
	0.0574	0.0438	−0.1619	0.6995	−0.0535	2.47	0.11	0.0046
Female	0.0130	−0.0188	0.0045	−0.2243	0.0161	0.0468	−0.0944	
	−0.0766	−0.0311	0.0340	0.1682	−0.0404	3.29	0.14	0.0002
*Age Group 16–65 Years: Allergic Rhinitis*								
2007								
Male	0.0019	0.0071	−0.0121	−0.1463	0.0034	−0.0052	−0.0367	
	0.1029	−0.0014	0.0760	−0.3452	−0.0428	2.83	0.13	0.0012
*Age Group * 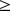 * 65 Years: Allergic Rhinitis*								
2011	0.0005	0.0000	0.0063	0.0115	−0.0012	0.0010	−0.0187	
female	−0.0375	0.0044	−0.0223	−0.3737	−0.0086	2.50	0.11	0.0042

For the 16–65 age group of outpatients, multiple linear regression model shows only results for male outpatients in the 2007 data are statistically significant (*P* = 0.0012).In this case, 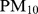, 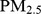, NO, and temperature positively predicts the increase of outpatient visits. Rainfall rates and relative humidity are negatively associated with outpatient visits.

For outpatients with ages greater than 66, statistically significant association between outpatient visits and air pollutants and meteorological factors occur mostly in the 2011 data for female outpatients (*P* = 0.0042). Positive increase in air pollutants 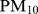, 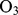, CO, and 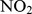 are associated with increase in outpatient visits. Temperature and relative humidity are negatively associated with the outpatient visits.

Table 6 shows multiple linear regression analysis of outpatient visits for asthma. For the 0–15 age group of both male and female outpatients, statistically significant associations occur only in the 2008 datat (*P* = 0.0015 for male, and *P* = 0.0005 for female). Air pollutants NO and 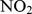 are positively associated with both male and female outpatient visits. Temperature and relative humidity are negatively associated with outpatient visits. Male outpatient visits are also positively associated with 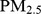 concentrations, while female outpatient visits are positively associated with 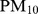 concentrations.

**Table 6:** Multiple Linear Regression Analysis of Asthma Outpatient Visits Regression Coefficients, *F* value, 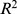, and *P* Value.

Sex	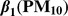	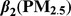	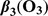	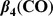	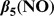	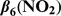	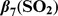	
	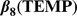	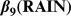	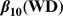	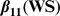	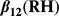	*F*-value	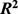	*P*-value
*Age Group 0–15 Years: Asthma*								
2008								
male	−0.0081	0.0062	−0.0206	−0.5042	0.0021	0.0727	−0.0601	
	−0.1523	−0.0239	0.0321	0.1445	−0.0221	2.76	0.12	0.0015
female	0.0058	−0.0060	−0.0039	−0.6655	0.0088	0.0283	−0.0627	
	−0.0724	0.0049	−0.0661	−0.6085	−0.0066	3.03	0.13	0.0005
*Age Group 16–65 Years: Asthma*								
2007								
Male	−0.0039	−0.0048	0.0019	−0.5143	0.0073	0.0093	0.1561	
	−0.1279	−0.0100	−0.0054	0.0547	0.0281	3.57	0.15	0.0001
2009								
male	0.0088	−0.0196	−0.0039	−0.1584	0.0035	0.0386	−0.0590	
	−0.0883	−0.0266	−0.0385	−0.6217	−0.0075	2.65	0.12	0.0024
female	0.0161	−0.0223	−0.0065	−0.4537	−0.0003	0.0830	−0.0815	
	0.0120	−0.0141	−0.0188	0.4914	0.0181	2.16	0.10	0.0144
2011								
female	0.0219	0.0000	0.0269	−0.1364	0.0190	−0.0300	−0.0559	
	−0.3124	−0.0115	−0.0740	−1.2689	−0.0007	4.41	0.18	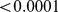
*Age Group * 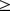 * 66 Years: Asthma*								
2007								
male	−0.0035	−0.0098	−0.0012	−0.3204	0.0144	−0.0005	0.0186	
	−0.1591	−0.0087	0.0711	0.0971	−0.0272	3.46	0.15	0.0001
2008								
male	−0.0061	0.0006	−0.0124	−0.4596	0.0054	0.0436	−0.0681	
	0.0053	−0.0002	−0.0406	−0.2538	0.0348	2.27	0.10	0.0095
2009								
male	−0.0071	0.0043	0.0127	0.1547	−0.0018	0.0579	−0.0893	
	−0.0085	−0.0144	−0.0001	0.4906	0.0021	2.07	0.09	0.0197
2011								
female	0.0150	−0.0000	0.0001	0.0939	0.0085	−0.0091	−0.0231	
	−0.1084	−0.0107	−0.0163	−0.0868	−0.0195	3.28	0.14	0.0002

For the elder group of male outpatients (16–65 years old), statistically significant associations occur in 2007 (*P* = 0.0001) and 2009 (*P* = 0.0024). In these two years of data, both NO and 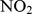 shows persistently positive while temperature shows persistently negative association with outpatient visits. For the female outpatients, statistically significant associations occur in the 2009 (*P* = 0.0144) and 2011 (P
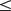
0.0001) data. 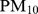 is the most significant factor that is persistently and positively associated with female outpatient visits.

For the eldest group of outpatients, statistically significant associations for male outpatients occur in the data of 2007 (*P* = 0.0001), 2008 (*P* = 0.0095), and 2009 (*P* = 0.0197). No pollutants show persistently positive associations with male outpatient visits. For the female outpatients, statistically significant association occur in the 2011 data (*P* = 0.0002). Air pollutants 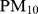, 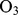, CO, and NO are positively associated with outpatient visits, while temperature and relative humidity are negatively associated with outpatient visits.

Table 7 shows multiple linear regression analysis of outpatient visits for pneumonia. For the age 0–15 group of male outpatients, statistically significant associations occur in the data of 2007 (P
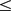
0.0001), 2009 (*P* = 0.0008), and 2011 (P
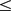
0.0001). Temperature and relative humidity exhibit persistently negative association with outpatient visits. For female outpatient, statistically siginificant associations occur in the 2007 (P
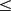
0.0001), 2009 (*P* = 0.0022), and 2011 (P
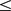
0.0001) data. 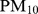 is the main pollutant that is persistently and positively associated with female outpatient visits. Temperatures and relative humidity are persistently and negatively associated with female outpatient visits.

**Table 7:** Multiple Linear Regression Analysis of Pneumonia Outpatient Visits Regression Coefficients, *F* value, 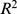, and *P* Value.

Sex	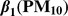	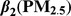	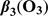	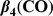	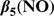	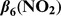	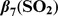	
	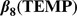	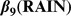	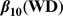	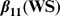	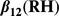	*F*-value	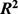	*P*-value
*Age Group 0–15 Years: Pneumonia*								
2007								
male	0.0084	−0.0232	−0.0108	0.8772	0.0094	−0.0120	0.1196	
	−0.5691	−0.0096	0.0415	−0.4870	−0.0301	5.59	0.22	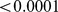
female	0.0045	−0.0179	−0.0167	1.0369	−0.0034	−0.0015	0.0209	
	−0.4374	0.0126	−0.0295	−0.5496	−0.1042	3.99	0.17	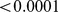
2009								
male	0.0294	−0.0275	−0.0028	−0.0945	−0.0127	0.0317	−0.2386	
	−0.0998	−0.0126	−0.1883	−0.4633	−0.0628	2.94	0.13	0.0008
female	0.0240	−0.0181	−0.0134	−0.2471	0.0102	0.0414	−0.0975	
	−0.0694	−0.0146	−0.0756	−0.5318	−0.0763	2.67	0.12	0.0022
2011								
male	−0.0022	−0.0000	0.0535	−1.5489	0.0402	0.0135	0.1126	
	−0.3419	−0.0389	−0.3596	−2.0619	−0.0283	5.83	0.23	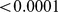
female	0.0032	−0.0000	0.0584	−0.6388	−0.0009	−0.0223	0.0266	
	−0.4399	−0.0604	−0.2782	−1.9063	−0.0325	9.66	0.33	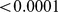
*Age Group 16–65 Years: Pneumonia*								
2007								
male	−0.0099	0.0057	−0.0072	0.1920	−0.0012	0.0176	0.0018	
	−0.0859	−0.0034	0.0099	−0.0949	−0.0049	3.58	0.16	0.0001
female	−0.0013	−0.0043	−0.0122	−0.0286	0.0018	0.0083	−0.0126	
	−0.0555	−0.0028	−0.0104	−0.2840	−0.0271	2.45	0.11	0.0051
2011								
male	0.0045	0.0000	−0.0028	−0.3053	0.0080	0.0117	0.0074	
	−0.0648	−0.0182	0.0230	−0.0491	0.0063	3.04	0.13	0.0005
female	0.0018	0.0000	0.0004	0.1477	0.0058	0.0220	−0.0376	
	−0.1375	−0.0157	0.0401	−0.0271	−0.0260	6.18	0.24	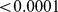

For the age 16–65 group of outpatients, statistically significant associations for male outpatients occur in the data of 2007 (*P* = 0.0001) and 2011 (*P* = 0.0005). Air pollutants 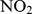 and 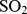 show persistently positive associations with male outpatient visits. Temperatures are negatively associated with male outpatient visits. For the female outpatients, statistically significant association occur in the 2007 (*P* = 0.0051) and 2011 (P
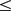
0.0001) data. Air pollutants CO, NO, and 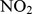 are positively associated with outpatient visits, while temperature and relative humidity are negatively associated with outpatient visits. For the eldest group of outpatients, no statistically significant associations are found for outpatient visits during the 2007–2011 data.

Table 8 shows multiple linear regression anaysis of associations of outpatient visits for COPD with respect to air pollutants and meteorological factors. Statistically significant association for the age 0–15 group of male outpatients occur only in the 2008 data (*P* = 0.0013). In this case, 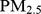, NO and 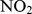 are positively while temperature and relative humidity are negatively associated with outpatient visits. Statistically significant associations for female outpatients also occur only in the 2008 data (*P* = 0.0007). Female COPD outpatients are positively associated with 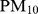, NO, and 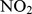; and negatively associated with temperature and relative humidity.

**Table 8:** Multiple Linear Regression Analysis of COPD Outpatient Visits Regression Coefficients, *F* value, 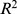, and *P* Value.

Sex	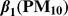	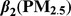	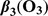	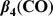	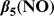	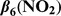	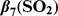	
	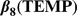	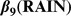	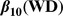	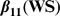	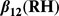	*F*-value	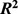	*P*-value
*Age Group 0–15 Years: COPD*								
2008								
male	−0.0079	0.0053	−0.0221	−0.5171	0.0019	0.0755	−0.0631	
	−0.1512	−0.0231	0.0317	0.1388	−0.0210	2.82	0.12	0.0013
female	0.0059	−0.0064	−0.0042	−0.6612	0.0091	0.0299	−0.0638	
	−0.0704	0.0055	−0.0644	−0.6309	−0.0075	2.97	0.13	0.0007
*Age Group 16–65 Years: COPD*								
2007								
male	0.0167	0.0030	0.0425	−1.4398	0.0228	0.0590	0.0863	
	0.0688	−0.0236	0.0489	0.0075	0.1220	2.21	0.10	0.0120
2008								
male	0.0237	−0.0422	−0.0388	−0.4829	0.0065	0.0969	−0.0591	
	0.0243	−0.0412	−0.0228	−0.0512	0.0374	2.22	0.10	0.0116
2009								
male	0.0099	−0.0325	0.0017	−0.1240	0.0032	0.0853	−0.0886	
	−0.0958	−0.0217	−0.1388	0.2359	−0.0222	2.29	0.10	0.0088
female	0.0250	−0.0514	0.0260	−0.7680	0.0194	0.0899	−0.0951	
	0.0065	−0.0195	−0.0727	1.3777	0.0340	2.78	0.12	0.0014
2011								
male	0.0095	−0.0000	0.0336	0.3874	−0.0102	−0.0060	−0.1294	
	−0.2667	−0.0248	−0.0140	−1.5874	−0.0040	2.42	0.11	0.0056
female	0.0305	0.0000	0.0320	−0.1361	0.0202	−0.0001	−0.1247	
	−0.3681	−0.0008	0.0194	−1.2646	0.0115	3.78	0.16	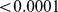
*Age Group * 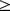 *65 Years: COPD*								
2008								
male	−0.0112	0.0060	−0.0206	−0.8232	0.0129	0.0635	−0.0550	
	0.0149	−0.0087	−0.0757	−0.4737	0.0335	2.22	0.10	0.0116
2010								
female	0.0086	0.0137	−0.0022	0.3628	−0.0045	0.0006	−0.0399	
	−0.0599	0.0034	0.0160	−0.2328	0.0185	3.45	0.15	0.0001
2011								
female	0.0182	0.0000	−0.0028	0.2111	0.0068	0.0210	−0.0641	
	−0.1424	−0.0027	0.0364	−0.0093	−0.0240	4.37	0.18	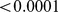

For the age 16–65 group of male outpatients, statististically siginificant associations occur in the 2007 (*P* = 0.0120), 2008 (*P* = 0.0116), 2009 (*P* = 0.0088), and 2011 (*P* = 0.0056). In these four years of data, COPD male outpatients are persistently and positively associated with 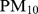. For femal COPD outpaitents, statistically significant associations occur in the 2009 (*P* = 0.0014) and 2011 (P
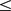
0.0001) data. 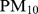, 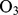, NO, and relative humidity are positively associated with female COPD outpatient visits.

For the eldest group of male outpatients (age greater than 66 years old), statistically significant associations occur only in the 2008 (*P* = 0.0116) data. In this case, 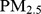, NO, 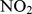, temperature, and relative humidity are positively associated with outpatient visits. For female outpatients, statistically significant associations occur in the 2010 (*P* = 0.0001) and 2011 (P
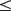
0.0001) data. In these two years of data, female COPD outpatients are positively assoicated with 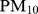, CO, and 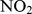. Temperatures are negatively associated with these outpatient visits.

## Summary

Taiwan contains high density of industrial factories, polluted air, and a comprehensive National Health Insurance program that covers population and contracted 92.1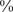 of all hospitals and clinics in Taiwan. In this work we used outpatient data from a hospital in a heavily industrial area in Taiwan to study the association between public health and air pollution in the context of a heavily polluted atmospheric environment during the period 2007–2011. We used hospital visit data from the Landseed Hospital, and air pollution and meteorological data from EPA to study the association between air pollution levels, meteorology, and hospital visit frequency for the period 2007–2011 over Chung-Li area. We test the normality of each data set, control for the confounding factors, and calculate correlation coefficient between the outpatient visits and air pollution and meteorology, and use multiple linear regression analysis to seek significance of these associations.

Table 9 shows a summary of list of main pollutants and meteorological factors that are statistically significant in associating with the respiratory diseases from multiple linear regression analysis. Temperature and relative humidity tend to be negatively associated with respiratory diseases. NO and 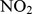 are two main air pollutants that are positively associated with respiratory diseases, followed by 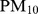, 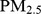, 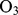, CO, and 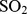. Young outpatients (age 0–15 years) are most sensitive to changing air pollution and meteorology factors, followed by the eldest (age 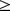
66 years) and age 16–65 years of outpatients. Outpatients for COPD diseases are most sensitive to air pollution and meteorology factors, followed by allergic rhinitis, asthma, and pneumonia diseases. In the context of sex difference to air pollution and meteorological factors, male outpatients are more sensitive than female outpatients in the 16–65 age groups, while female outpatients are more sensitive than male outpatients in the young 0–15 age groups and in the eldest age groups. In total, female outpatients are more sensitive to air pollution and meteorological factors than male outpatients.

**Table 9:** Summary of Multiple Linear Regression Analysis of Outpatient Visits for Respiratory Diseases.

	Allergic Rhinitis	Asthma	Pneumonia	COPD
*0–15 Age Group*				
male	+: NO, WS	+: 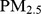 , NO, 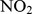		+: 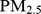 , NO, 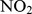
	−: RH	−: T, RH	−: T, RH	−: T, RH
female	+: 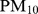 , 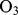 , NO, 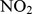	+ 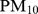 , NO, 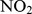	+: 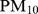	+: 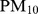 , NO, 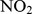
	−: T, RH	−: T, RH	−: T, RH	−: T, RH
*16–65 Age Group*				
male	+: 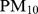 , 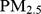 , NO, T	+: NO, 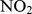	+: 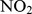 , 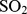	+: 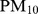
	−: RH	−: T	−: T	
female		+ 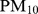	+: CO, NO, 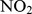	+: 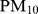 , 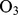 , NO
			−: T, RH	
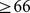 *Age Group*				
male				+: 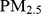 , NO, 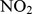
				+: T, RH
female	+: 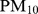 , 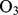 , CO, 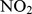	+: 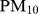 , 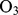 , CO, NO		+: 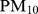 , CO, 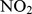
	−: T, RH	−: T, RH		−: T

+ means positive association; − means negative association; T: temperature; RH: relative humidity; WS: wind speed.
